# Comprehensive analysis of the cuproptosis-related gene DLD across cancers: A potential prognostic and immunotherapeutic target

**DOI:** 10.3389/fphar.2023.1111462

**Published:** 2023-04-03

**Authors:** Weiguang Yang, Qiang Guo, Haiyang Wu, Linjian Tong, Jian Xiao, Yulin Wang, Rui Liu, Lixia Xu, Hua Yan, Zhiming Sun

**Affiliations:** ^1^ Clinical College of Neurology, Neurosurgery and Neurorehabilitation, Tianjin Medical University, Tianjin, China; ^2^ Department of Graduate School, Tianjin Medical University, Tianjin, China; ^3^ Department of Orthopaedics, Baodi Clinical College of Tianjin Medical University, Tianjin, China; ^4^ Duke Molecular Physiology Institute, Duke University School of Medicine, Duke University, Durham, NC, United States; ^5^ Tianjin Key Laboratory of Cerebral Vascular and Neurodegenerative Diseases, Tianjin Neurosurgical Institute, Tianjin Huanhu Hospital, Tianjin, China; ^6^ Department of Orthopaedics, Tianjin Huanhu Hospital, Tianjin, China

**Keywords:** dld, prognosis, cuproptosis, immune cell infiltration, bioinformatics analysis, pan-cancer

## Abstract

DLD is a key gene involved in “cuproptosis,” but its roles in tumor progression and immunity remain unclear. Exploring the potential mechanisms and biological roles of DLD may provide new insights for therapeutic strategies for tumors. In the present study, we analyzed the role of DLD in a variety of tumors by using several bioinformatic tools. The results showed that compared with normal tissues, tumor tissues representing multiple cancers showed significant differential expression of DLD. High DLD expression was associated with a good prognosis in BRCA, KICH, and LUAD. Conversely, high expression levels of DLD were detrimental to patient prognosis in many other tumors, such as COAD, KIRC, and KIRP. In addition, the associations of DLD with infiltrating immune cells, genetic alterations and methylation levels across cancers were assessed. Aberrant expression of DLD was positively correlated with most infiltrating immune cells, especially neutrophils. The DLD methylation level was significantly decreased in COAD, LIHC, and LUSC but significantly increased in BRCA. DLD had the highest mutation rate (6.04%) in ESCA. In LUSC, patients with genetic alterations in DLD showed a poorer prognosis. At the single-cell level, the roles of DLD in regulating cancer-associated biological functions, such as metastasis, inflammation, and differentiation, were explored. Afterward, we further investigated whether several disease-associated genes could be correlated with DLD. GO enrichment analysis indicated that DLD-related genes were mainly associated with mitochondria-related cellular components, aerobic respiration and the tricarboxylic acid cycle. Finally, the correlations between DLD expression and immunomodulatory genes, immune checkpoints, and sensitivity to some antitumor drugs were investigated. It is worth noting that DLD expression was positively correlated with immune checkpoint genes and immunomodulatory genes in most cancers. In conclusion, this study comprehensively analyzed the differential expression, prognostic value and immune cell infiltration-related function of DLD across cancers. Our results suggest that DLD has great potential to serve as a candidate marker for pancancer prognosis and immunotherapy and may provide a new direction for cancer treatment development.

## Introduction

Cancer is one of the leading diseases causing human death and a significant medical burden worldwide. According to epidemiological surveys, lung cancer (828,100; 20.4%), colorectal cancer (408,000; 10.0%) and gastric cancer (396,500; 9.8%) are the three most common tumors worldwide, and the three deadliest cancers are lung cancer (657,000; 27.2%), liver cancer (336,400; 13.9%) and stomach cancer (288,500; 12.0%) (Maomao et al., 2022). The properties of tumors include biological behaviors acquired during their development, such as evasion of growth inhibitors, resistance to cell death, and achievement of replicative immortality (Hanahan and Weinberg, 2011). Tumor occurrence and development are complex multifactorial processes driven by genetic variation: carcinogenesis is promoted and developed, and carcinogenic components change and induce proliferation (Xiao et al., 2022). Thus, the development of more effective approaches for the early detection and treatment of tumors will help reduce cancer mortality (Bray et al., 2018; Cheng et al., 2022). Pancancer genomic analyses may be helpful for discovering new cancer-associated genes and elucidating their functions to further understand the incredibly complicated process of carcinogenesis (ICGC/TCGA Pan-Cancer Analysis of Whole Genomes Consortium, 2020).

There are many patterns of cell death, such as necroptosis, ferroptosis, and pyroptosis. Of note, a new atypical form of cell death, cuproptosis, was recently identified. Several studies have found that increased amounts of copper ions could bind acylated lipid elements and block the TCA cycle, resulting in the aggregation and destruction of Fe-S cluster proteins, which leads to macromolecular cytotoxic stress and death (Chen et al., 2022; Cobine and Brady, 2022; Jiang et al., 2022). Moreover, copper ions are dispersed extensively throughout different tissues, and the level of copper ions is managed by a set of proteins, such as copper chaperones and Cu transporters. Previous studies have concluded that copper ions perform fundamental roles in energy metabolism, signal transduction, iron uptake and other important biological processes. Dysregulation of copper ions may lead to abnormal biological processes, which in turn cause various diseases, including anemia, thrombocytopenia, and cancer (Tsvetkov et al., 2022; Yang et al., 2022). This novel cell death mechanism may provide a novel therapeutic idea for cancers.

Dihydrolipoamide dehydrogenase (DLD) is an important enzyme involved in the complexes that make up α-ketoglutarate dehydrogenase, α-ketoadipate dehydrogenase and glycine decarboxylase (Duarte et al., 2021). It is also involved in the decarboxylation of pyruvate, the product of which is converted into acetyl coenzyme A in the tricarboxylic acid (TCA) cycle. Consistently, DLD encodes the moonlight protein, which can adhere to the surface of metal oxides. As a homodimeric flavin-dependent enzyme, DLD could affect the oxidation of dihydrolipoamide. When exerting redox activity, it could also generate reactive oxygen species (ROS), which are involved in the apoptotic process and therefore can act as exogenous anticancer agents (Fleminger and Dayan, 2021; Wu et al., 2021). In this study, the expression of DLD in multiple cancers was analyzed using a combination of various bioinformatic tools, such as differential gene expression level analysis, protein level expression analysis, and survival analysis. Additionally, the correlations of DLD expression with infiltrating immune cells, immunoregulatory genes and drug sensitivity were explored. The results showed that DLD is not only a biomarker closely related to prognosis in several cancers but also strongly correlated with immunomodulation and antitumor drug sensitivity.

## Materials and methods

### DLD expression level analysis

TIMER2.0 (Tumor Immune Estimation Resource, version 2, http://timer.cistrome.org/) (Li et al., 2020) and GEPIA2.0 (Gene Expression Profiling Interactive Analysis, version 2, http://gepia2.cancer-pku.cn/#analysis) (Tang et al., 2017) were used to compare the expression levels of DLD between normal and neoplastic tissues at corresponding sites. The expression levels of DLD genes are expressed using a log2 (TPM+1) scale, where TPM represents per million transcripts. TIMER2.0 analysis was based on data from The Cancer Genome Atlas (TCGA). GEPIA2.0 analysis was based on data from the TCGA and a genotype-tissue expression (GTEx) dataset. For GEPIA2.0, the *p*-value cutoff was 0.05, and the |Log2FC| cutoff was 1. The relationship between tumor pathological stage and DLD expression was analyzed with this bioinformatic tool. Then, the protein expression and DNA methylation of DLD were analyzed in the Clinical Proteomic Tumor Analysis Consortium (CPTAC) dataset through the UALCAN (http://ualcan.path.uab.edu/analysis-prot.html) platform (Chen et al., 2019; Chandrashekar et al., 2022). Information on the number of GEPIA2.0 samples is shown in Supplementary Table S1.

### Immunohistochemical (IHC) staining

IHC staining images of the DLD protein in seven tumor tissues and corresponding normal tissues were obtained from the HPA (Human Protein Atlas, http://www.proteinatlas.org/) to further confirm the differences in DLD expression at the protein level. In addition, we collected paraffin-embedded tissue sections from 12 patients with glioma from the Department of Pathology, Tianjin Huanhu Hospital. These sections were analyzed with reference to previous studies (Yang et al., 2018; Huang et al., 2019). The antibody used was Anti-Lipoamide Dehydogenase (1:150, Abcem, ab124926, Cambridge, United Kingdom). Subsequently, Fiji software was used to assess the mean optical density values and the percentage of positive area of the images (Schindelin et al., 2012). The clinical information of glioma patients are included in Supplementary Table S9.

### Survival analysis

GEPIA 2.0 was used to explore the prognostic value of DLD, including the value for predicting overall survival (OS) and disease-free survival (DFS), for all types of tumors. Cox regression analysis was performed to determine whether DLD expression was associated with OS or DFS. Survival analysis was conducted using the Kaplan‒Meier method, and the median value was applied as the cutoff to divide samples into high and low expression groups. Survival curve hypothesis testing was performed by the log-rank test. *p*-value < 0.05 was considered significant.

### Gene mutation analysis

The cBioPortal (https://www.cbioportal.org/) tool was used to analyze the mutational status of DLD, including mutation frequency, mutation type, and mutation site information, in all TCGA tumor subtypes. Survival data, including OS, DFS, disease-specific survival (DSS), and progression-free survival (PFS) results, were then compared across all TCGA cancer types. Information regarding DLD mutations is summarized in Supplementary Table S2. Patient characteristics for the DLD mutation analyses are included in Supplementary Table S3.

### Immune cell infiltration analysis

The TIMER 2.0 tool was used to investigate the relationship between DLD expression and immune cell infiltration. The following algorithms were chosen for this analysis: TIMER, EPIC, QUANTISEQ, MCPCOUNTER and XCELL.

### Single-cell sequencing analysis

The CancerSEA (http://biocc.hrbmu.edu.cn/CancerSEA/home.jsp) tool was used to explore the correlations between DLD expression and different tumor biological functions at the single-cell level (Yuan et al., 2019). Based on the conditions of *p-value* < 0.01 and correlation>0.3, we obtained different biological functions with significant results in five tumors. The correlations and corresponding *p value*s of cancer types and biological functions are shown in Supplementary Table S4. The distribution of DLD expression in different cancers is presented by using t-distribution random neighborhood embedding (t-SNE) plots.

### Functional enrichment analysis of DLD-related genes

The STRING tool (https://string-db.org/) was used for DLD-related protein‒protein interaction analysis (Franceschini et al., 2013). Then, the top 100 DLD-associated genes were obtained and subjected to Pearson correlation analysis *via* GEPIA2 (Supplementary Table S5). Finally, GO and KEGG enrichment analyses were performed for these 100 genes by using the R package ClusterProfiler (version 3.14.3), and the results obtained are displayed in Supplementary Table S6 and Supplementary Table S7. The screening conditions were FDR<0.1 and *p-value* < 0.05.

### Statistical analysis

Analysis of the differential expression of DLD across cancers was performed by the Wilcoxon test or Student’s t-test. Correlation analysis between DLD expression and other variables was performed using the Spearman or Pearson test. *p*< 0.05 was considered significant.

## Results

### Differential expression analysis of DLD across cancers

**FIGURE 1:**
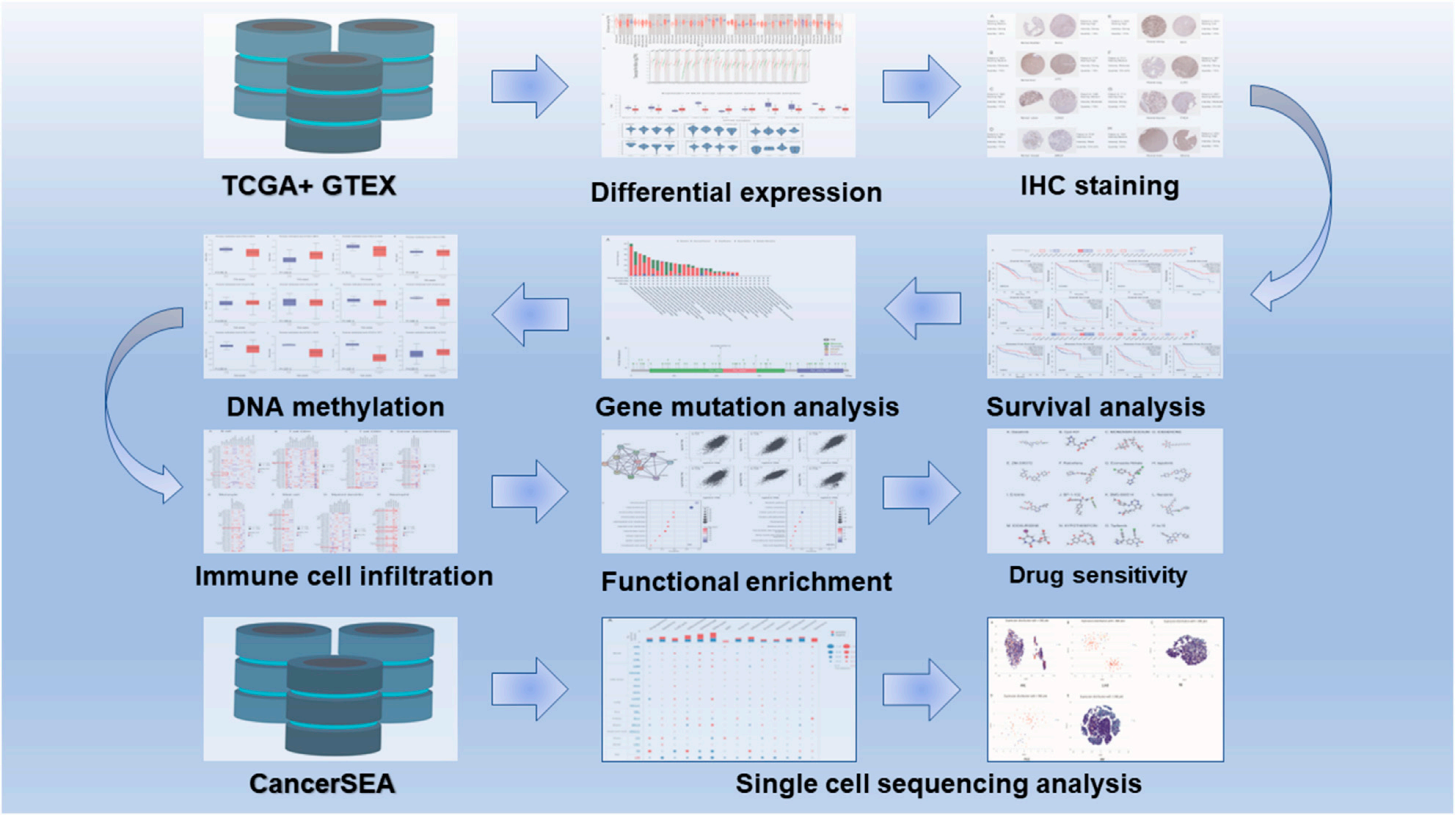
Flow chart for pancancer analysis of DLD expression.

**FIGURE 2:**
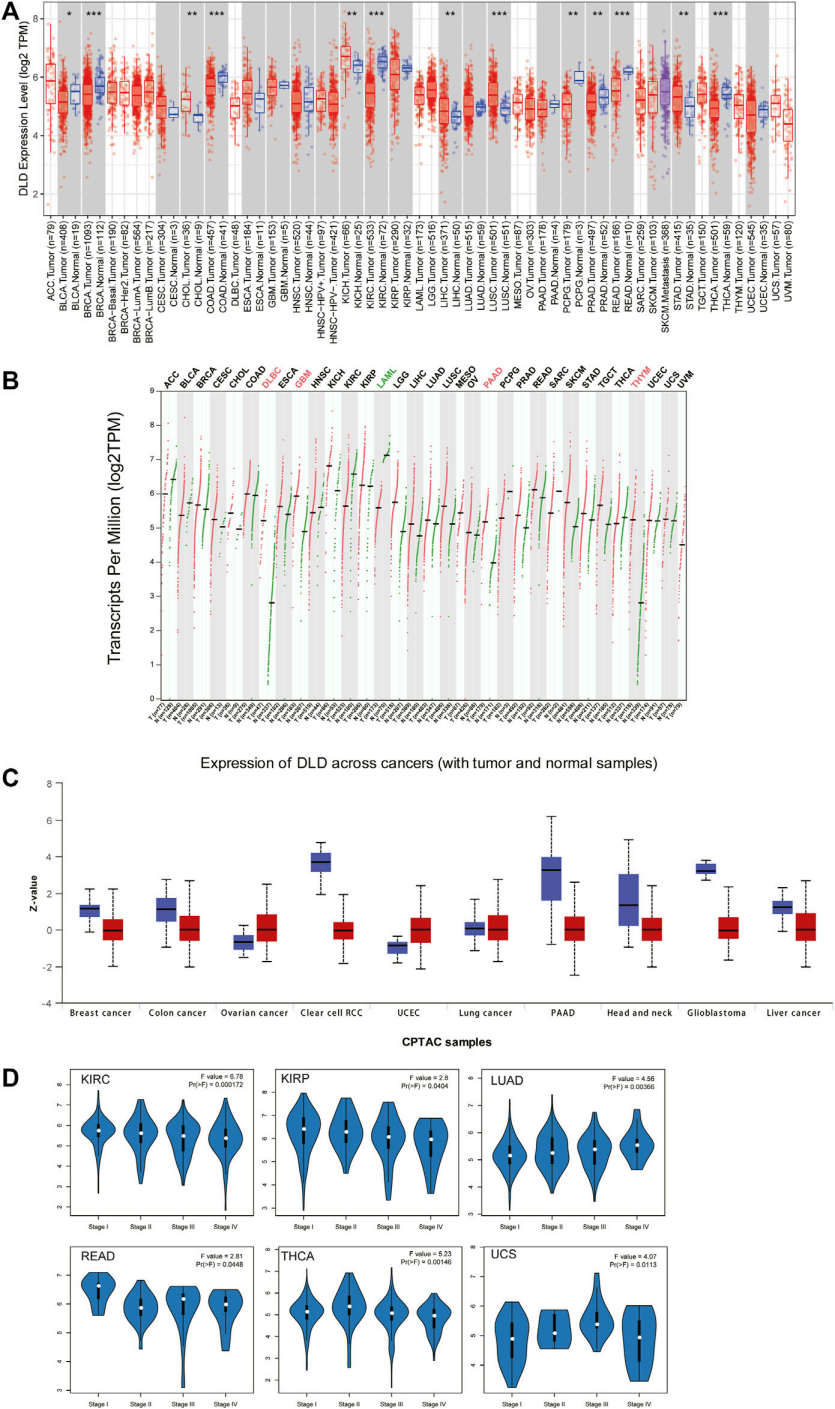
Differential expression of DLD across cancers. **(A)** mRNA level of DLD determined with the TIMER2.0 database. ***p < 0.001; **p < 0.01; *p < 0.05. **(B)** mRNA level of DLD determined with the GEPIA2.0 database. **(C)** Protein level of DLD determined with the CPTAC database. **(D)** Violin plots showing the relationship between the DLD expression level and pathological stage.

**FIGURE 3:**
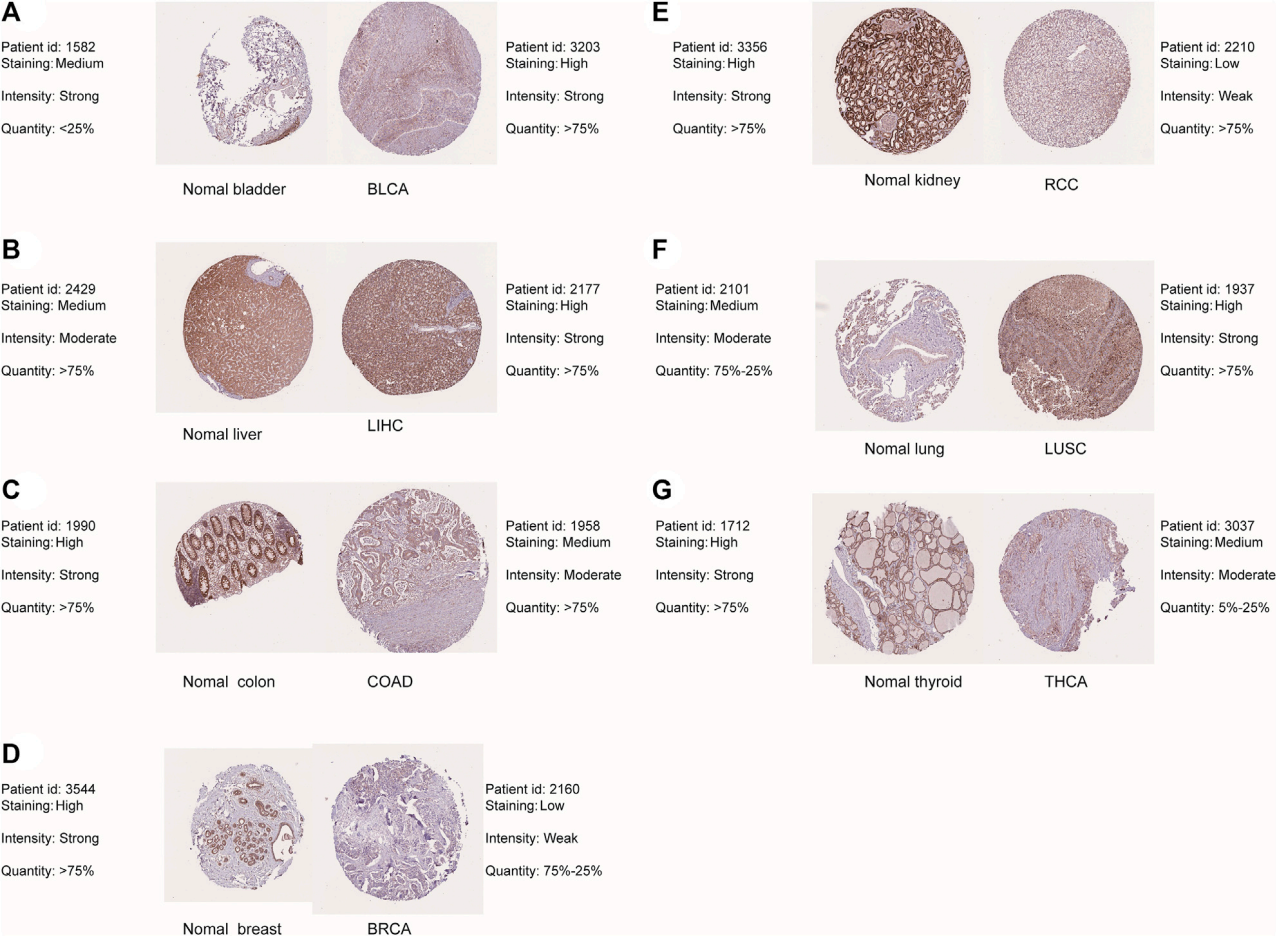
Differential expression of DLD between tumor tissues and corresponding normal tissues from the HPA database. **(A)** BLAC. **(B)** LIHC. **(C)** COAD. **(D)** BRCA. **(E)** RCC. **(F)** LUSC. **(G)** THCA.

The flow chart for the pancancer analysis of DLD expression is shown in Figure 1. As shown in Figure 2A, the expression level of DLD was downregulated in 8 tumor types including BLCA, BRCA, COAD, KIRC, PCPG, PRAD, READ, and THCA. In contrast, DLD expression was significantly upregulated in 5 tumors including CHOL, KICH, LIHC, LUSC, and STAD. For the results based on the data from the TCGA and GTEx evaluated with GEPIA2.0, we found that the expression level of DLD was upregulated in 4 cancer types including DLBC, GBM, PAAD, and THYM. Correspondingly, the DLD expression level was significantly downregulated in LAML (Figure 2B). Next, analysis of data from the CPTAC database showed that the DLD protein expression level differed in various cancers apart from lung cancer (Figure 2C). In addition, the violin plots in Figure 2D show the relationship between the DLD expression level and pathological stage. The results revealed that the DLD expression level was correlated with pathological stage in KIRC, KIRP, LUAD, READ, THCA, and UCS. No statistically significant results were found in other types of malignancies (Supplementary Figure S1). We also obtained IHC staining results from the HPA database and compared them with the results from the TIMER2.0 database. Similar results were found in both analyses. As displayed in Figures 3A–G, IHC analysis demonstrated moderate or high staining in normal bladder, breast, intestine, kidney, and thyroid tissues, corresponding to weak or moderate staining in the corresponding tumor tissues. In contrast, DLD staining was weak or moderate in normal lung and liver tissues but high in the corresponding tumor tissues.

### Survival analysis of DLD across cancers

**FIGURE 4:**
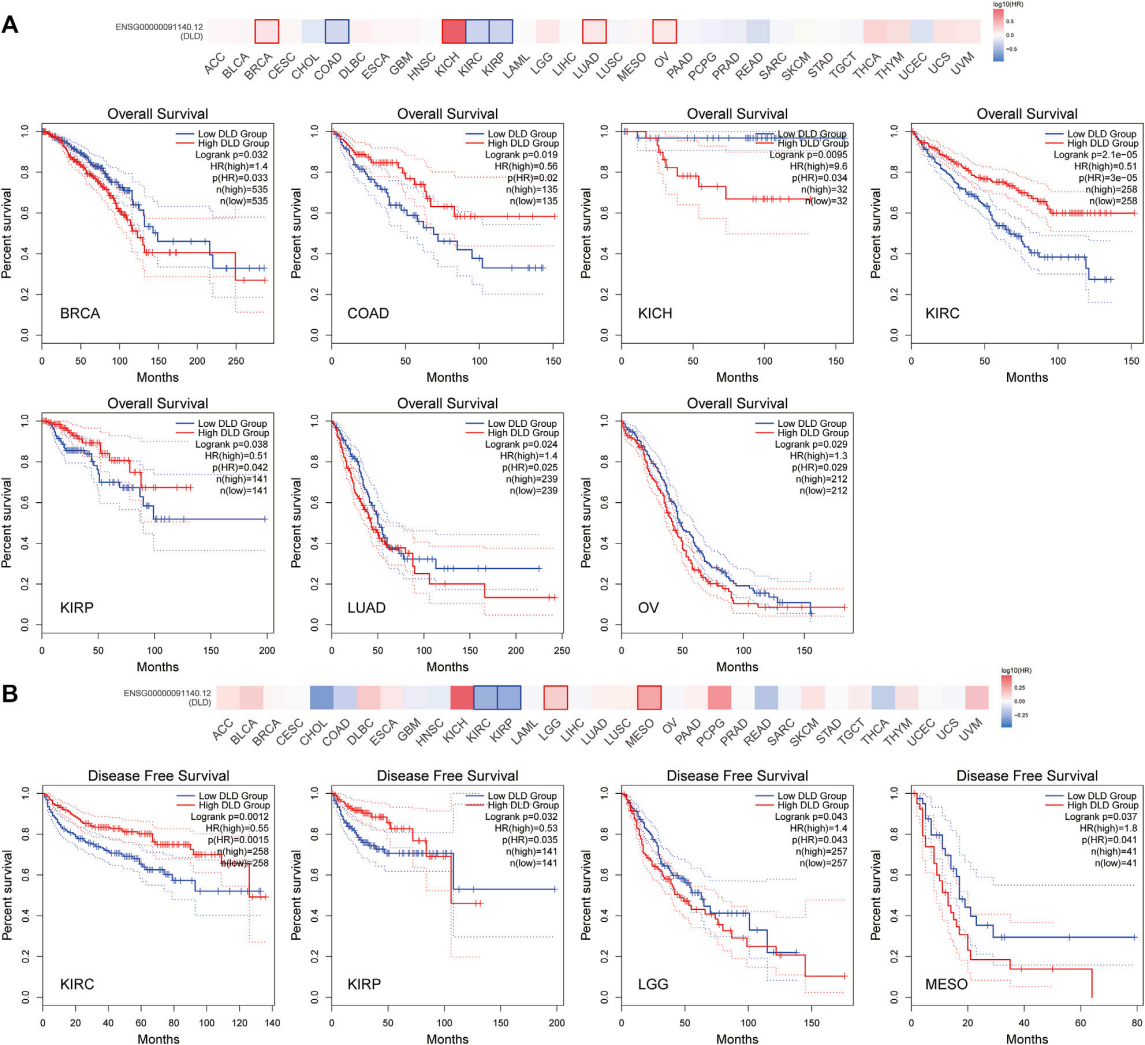
Survival analysis of DLD in pancancer data from the GEPIA2.0 database. **(A)** The impact of DLD expression on overall survival. **(B)** The impact of DLD expression on disease-free survival.

According to the expression level of DLD, samples from the TCGA and GTEx datasets were divided into high and low expression groups. The results in Figures 4A, B show that elevated levels of DLD expression were associated with poorer OS in patients with BRCA (*p* = 0.032, HR = 1.4), KICH (*p* = 0.0095, HR = 9.6), LUAD (*p* = 0.024, HR = 1.4) or OV (*p* = 0.029, HR = 1.3). In the other three tumors, COAD (*p* = 0.019, HR = 0.56), KIRC (*p* = 2.1e-05, HR = 0.51) and KIRP (*p* = 0.038, HR = 0.51), elevated expression levels of DLD were associated with better OS. With regard to PFS, elevated levels of DLD expression were associated with longer DFS in both tumor types (KIRC and KIRP). Opposite results were obtained for the two other cancers, LGG and MESO.

### Genetic alterations of DLD across cancers

**FIGURE 5:**
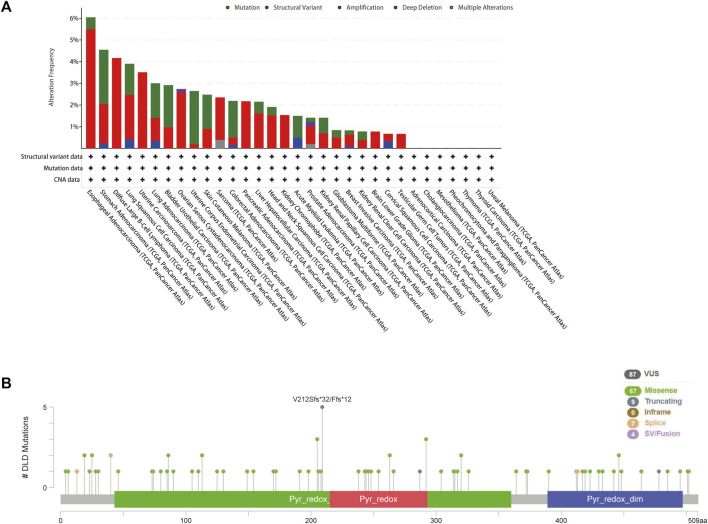
Genetic alterations of DLD in pancancer data from the cBioPortal tool. **(A)** The mutational status of DLD in each type of cancer. **(B)** The mutated site and main mutation types of DLD.

**FIGURE 6:**
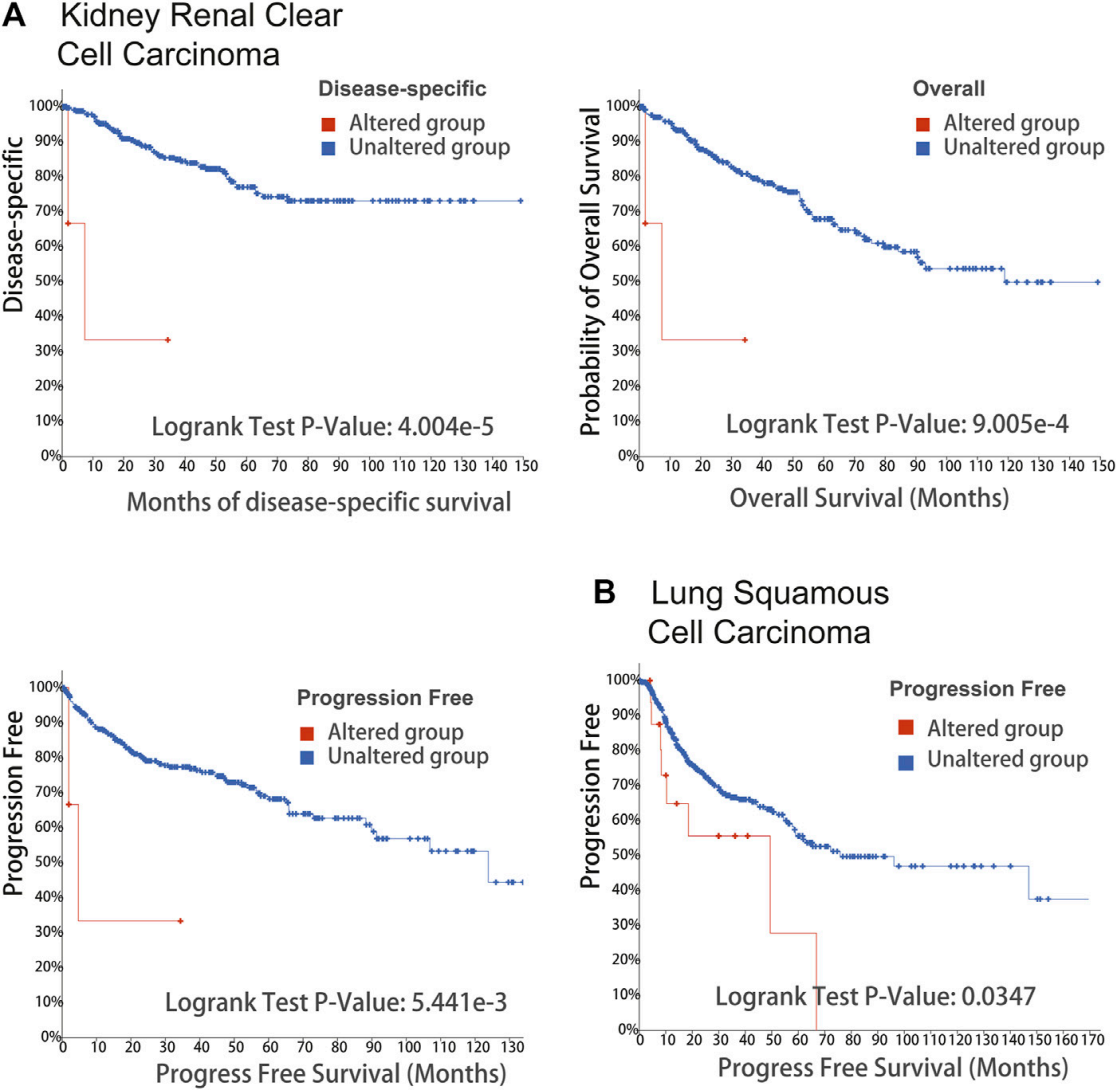
Survival analysis of DLD alterations in cancers. The correlation between DLD mutation and prognostic value is shown in **(A, B)** and was determined using cBioPortal (DSS, DFS, PFS, and OS).

The cBioPortal tool was used to explore the mutational status of DLD across cancers. We found that DLD had the highest mutation rate (6.04%) in ESCA. Among the evaluated mutation, the “Amplification” type had the highest rate at 5.49% (Figure 5A). In addition, “Mutation” was the main type of genetic alteration in DLD, mainly located in the “PDB1zy8” domain. A potentially clinically significant missense mutation, an alteration at the V212Sfs*32/Ffs*12 locus, was detected in 5 samples (Figure 5B). Subsequently, to further investigate the relationship between genetic alterations and prognosis, we performed survival analyses including OS, DFS, DSS, and PFS analyses. As shown in Figures 6A, B, patients suffering from KIRC with DLD mutations showed poorer DSS (*p* = 4.004e-5), OS (*p* = 9.005e-4), and PFS (*p* = 5.441e-3). In lung squamous cell carcinoma, patients with DLD genetic alterations showed poorer PFS as well (*p* = 0.0347).

### DNA methylation levels of DLD across cancers

**FIGURE 7:**
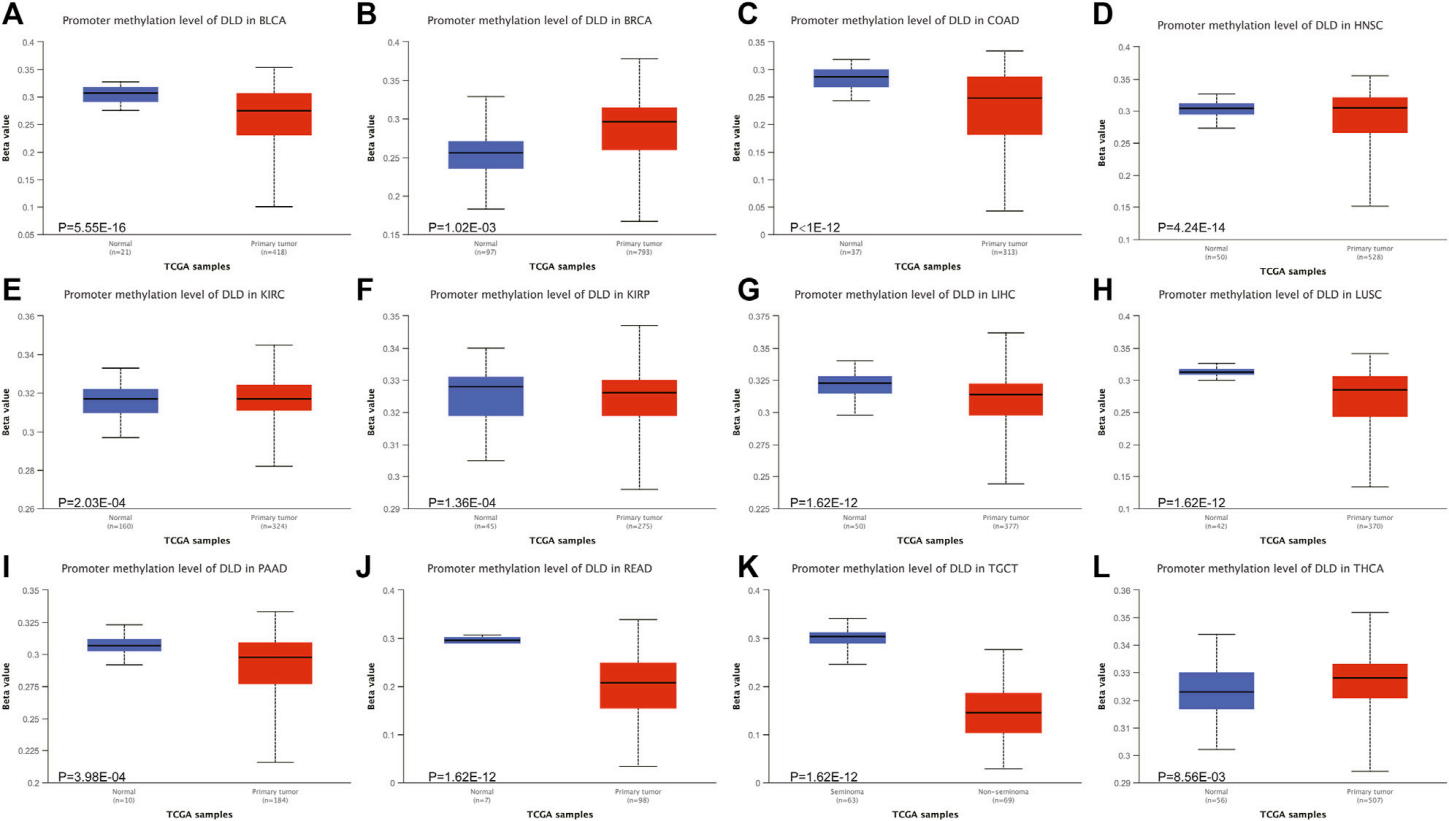
The DNA methylation levels of DLD in tumors. **(A–L)** The UALCAN database was used to compare the methylation of DLD between primary tumor samples and normal samples.

Epigenetic alterations, such as DNA methylation, could be part of the causal chain in cancer progression. Therefore, we explored the altered methylation levels of DLD in tumor tissues compared to normal tissues in pancancer data using the UALCAN tool. Our findings suggested that DLD methylation levels were significantly different in 12 tumor types (Figures 7A–L). Among these tumor types, BLCA, COAD, LIHC, LUSC, PAAD, READ, and TGCT showed significantly decreased DLD promoter methylation, while BRCA showed a significant increase. In the remaining types of tumors, no significant differences were observed in the methylation level of DLD (Supplementary Figure S2).

### Effect of DLD on immune cell infiltration

**FIGURE 8:**
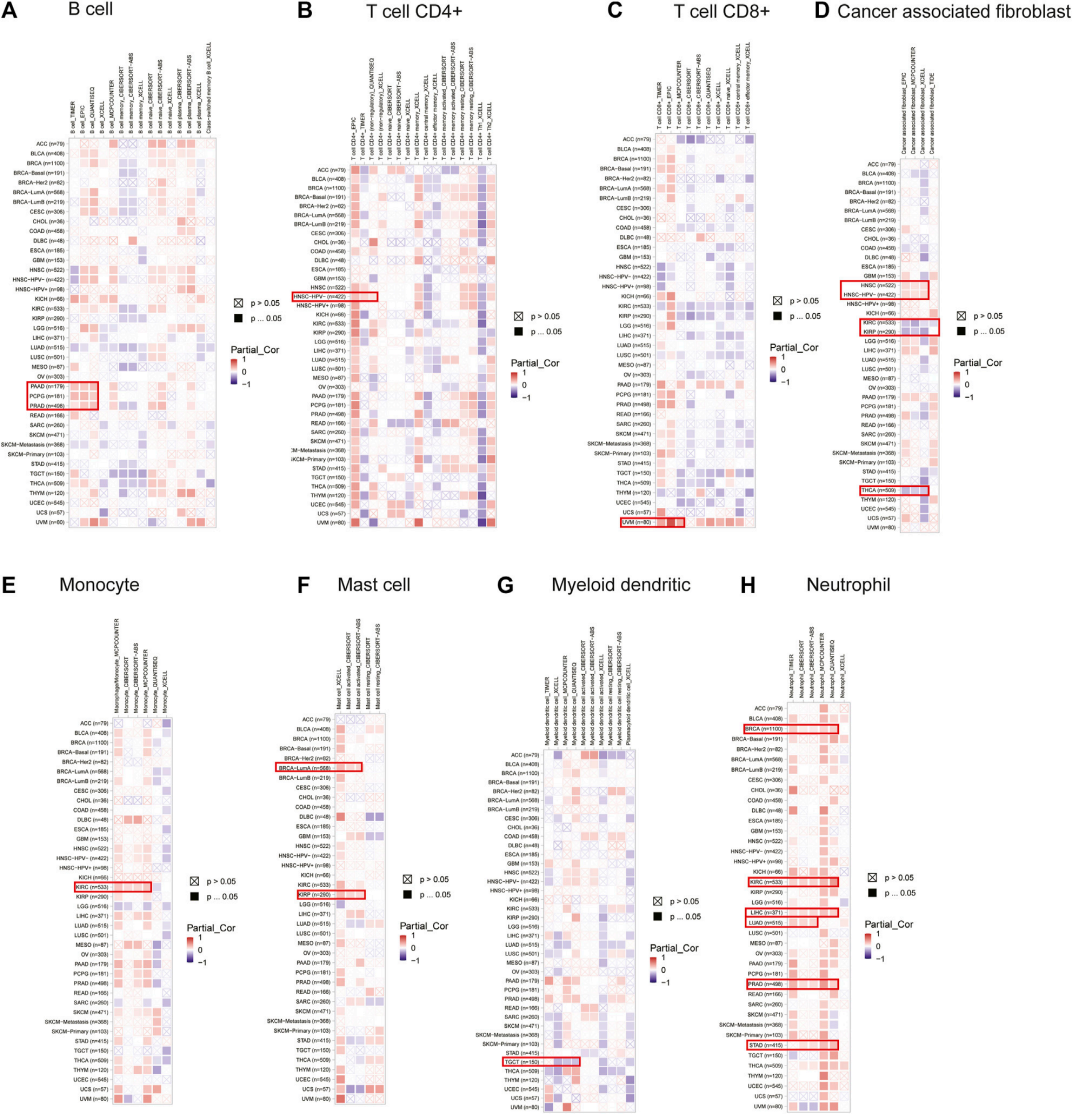
Correlations between the expression of DLD and immune cells in data from the TIMER2.0 database. The heatmap depicts the relationships between DLD and the infiltration of B cells **(A)**, CD4^+^ T cells **(B)**, CD8^+^ T cells **(C)**, cancer-associated fibroblasts **(D)**, monocytes **(E)**, mast cells **(F)**, myeloid dendritic cells **(G)** and neutrophils **(H)**.

The correlation between DLD expression and immune cell infiltration was analyzed by using the TIMER2.0 tool. Our results revealed that the expression of DLD was positively correlated with B-cell infiltration in PAAD, PCPG, and PRAD (Figure 8A); CD4^+^ T-cell infiltration in HNSC-HPV- (Figure 8B); CD8^+^ T-cell infiltration in UVM (Figure 8C); cancer-associated fibroblast infiltration in HNSC (Figure 8D); monocyte infiltration in KIRC (Figure 8E); mast cell infiltration in BRCA-LumA and KIRP (Figure 8F); and neutrophil infiltration in BRCA, KIRC, LIHC, LUAD, PRAD, and STAD (Figure 8H). Moreover, the expression of DLD was negatively correlated with myeloid dendritic cell infiltration in TGCT (Figure 8G) and cancer-associated fibroblast infiltration in KIRC, KIRP, and THCA (Figure 8D). However, as summarized in Supplementary Figures S3, S4, DLD expression was not significantly correlated with other tumor-infiltrating immune cells.

### Expression of DLD at the single-cell level and its relationship with tumor biological behavior

**FIGURE 9:**
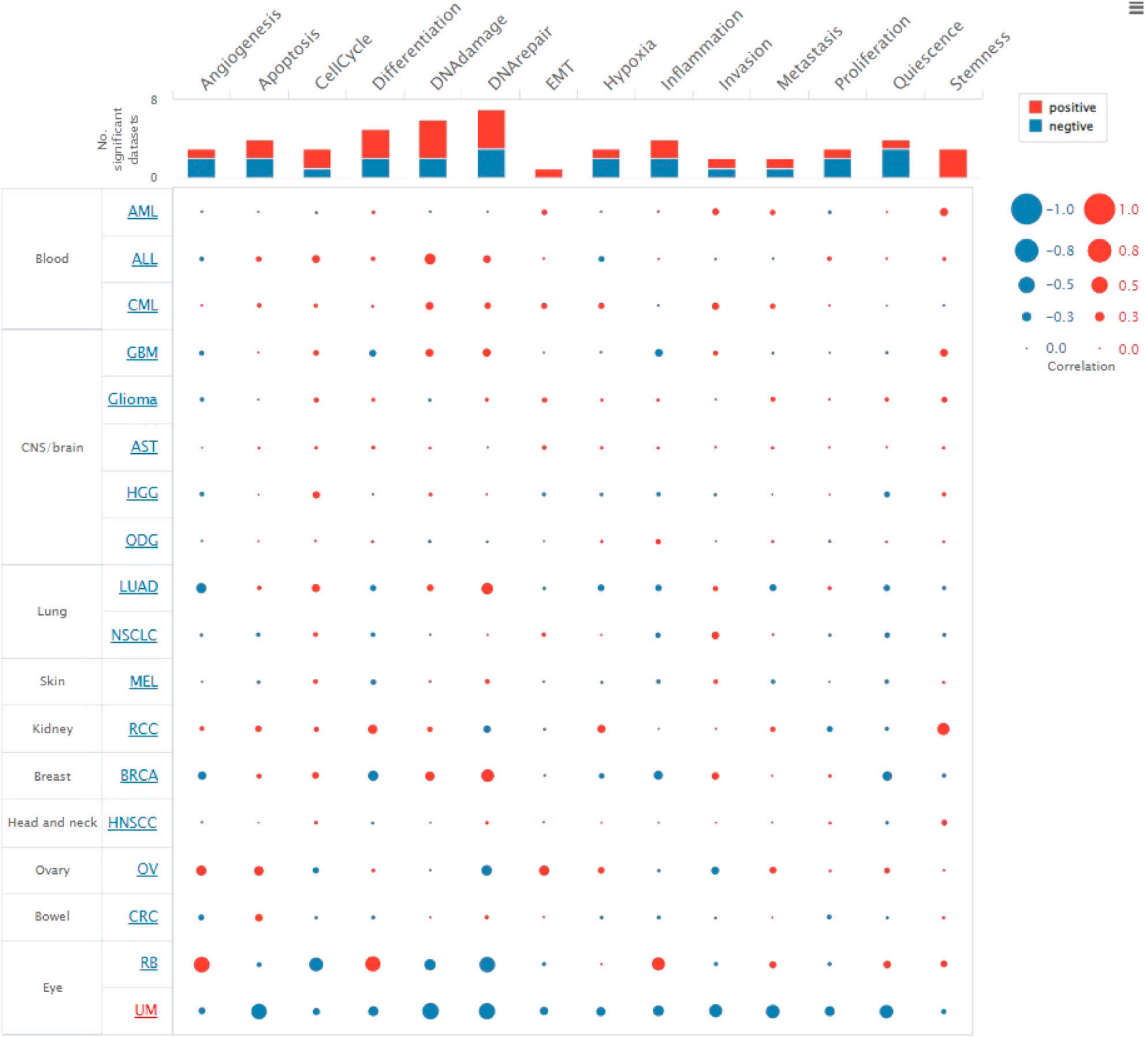
The correlations of 14 tumor biological functions with DLD.

**FIGURE 10:**
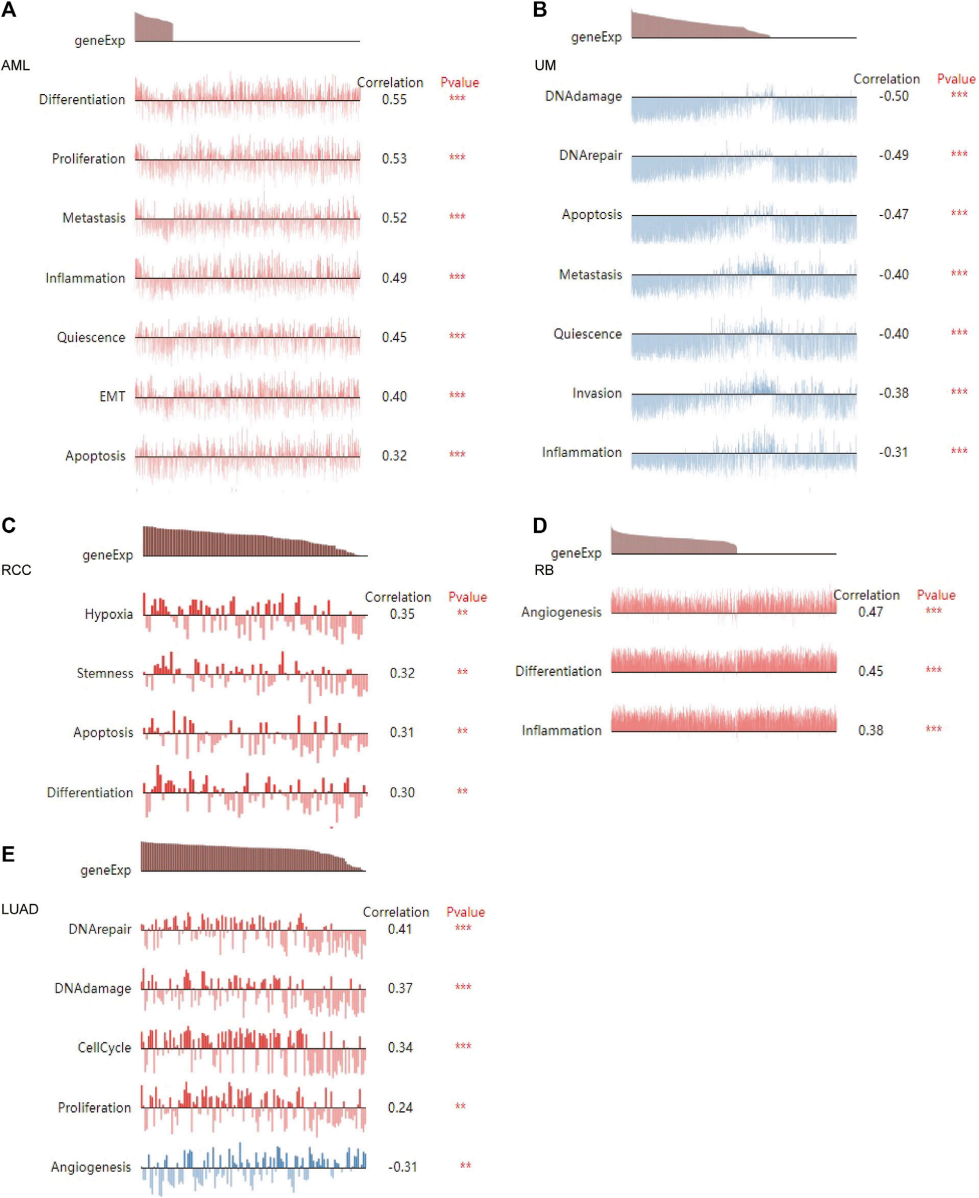
The relationship between DLD and tumor biological function in AML **(A)**, UM **(B)**, RCC **(C)**, RB **(D)**, and LUAD **(E)**. ***p < 0.001; **p < 0.01.

**FIGURE 11:**
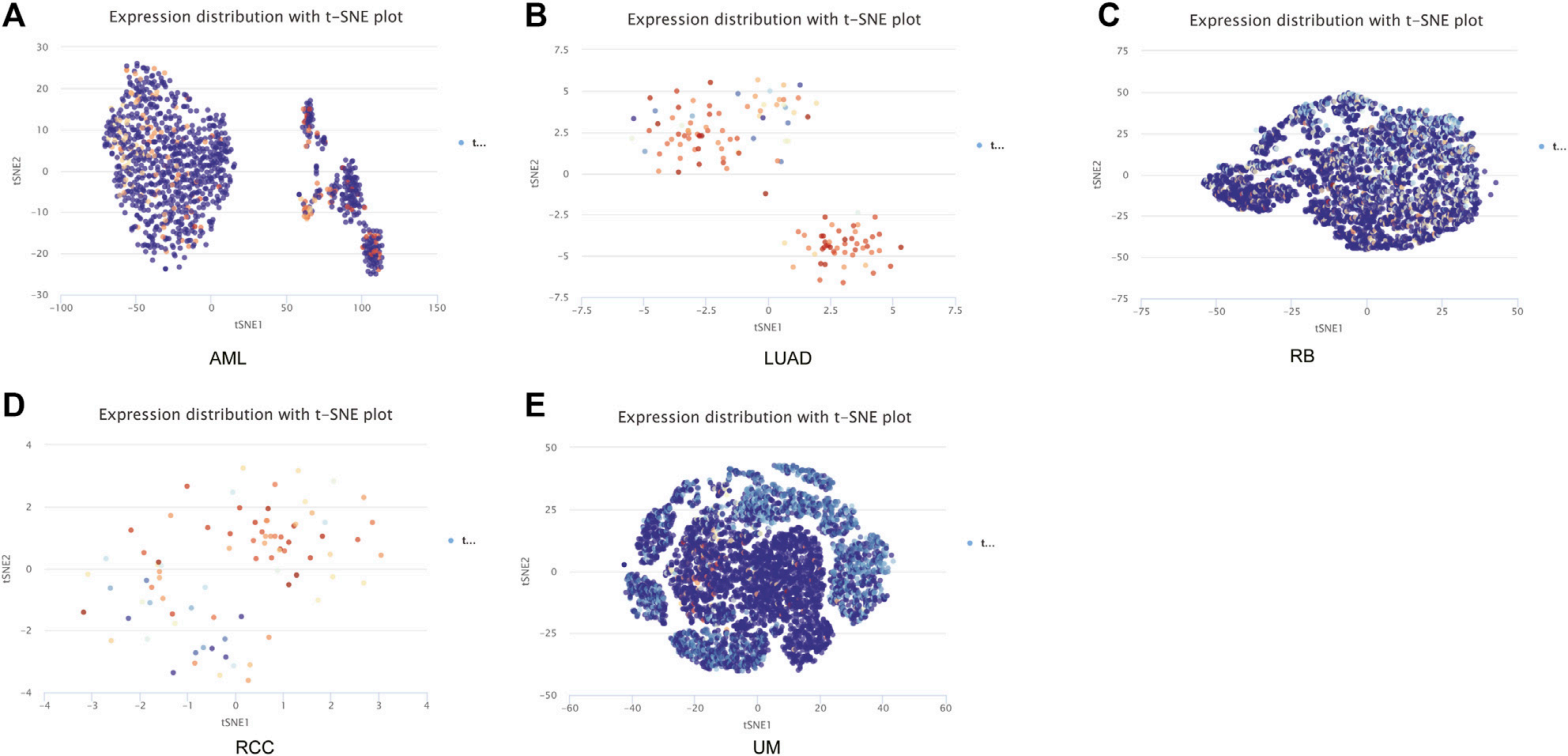
The distribution of DLD expression at the single-cell level in the above five tumors is demonstrated in t-SNE plots. **(A)** AML. **(B)** LUAD. **(C)** RB. **(D)** RCC. **(E)** UM.

The CancerSEA tool was used to analyze the expression level of DLD at the single-cell level and then further explore the relationships between DLD and different tumor biological functions across cancers. Figure 9A demonstrates the correlations of 14 tumor biological functions with DLD in multiple cancer types. In AML, DLD expression was positively correlated with 7 tumor biological behaviors including apoptosis, differentiation, EMT, inflammation, metastasis, proliferation, and quiescence (Figure 10A). With regard to UM, DLD expression was negatively correlated with 7 tumor biological behaviors including apoptosis, DNA damage, DNA repair, inflammation, invasion, metastasis, and quiescence (Figure 10B). In RCC, 4 biological behaviors including apoptosis, differentiation, hypoxia, and stemness were positively correlated with DLD expression (Figure 10C). For RB, biological functions such as angiogenesis, differentiation, and inflammation were positively correlated with DLD expression (Figure 10D). In LUAD, our findings suggested that DLD expression was positively correlated with 4 tumor biological behaviors including the cell cycle, DNA damage, DNA repair, and proliferation and negatively correlated with angiogenesis (Figure 10E). In addition, the distribution of DLD expression at the single-cell level in the above 5 tumor types is demonstrated in t-SNE plots shown in Figures 11A–E.

### DLD-related gene enrichment analysis

**FIGURE 12:**
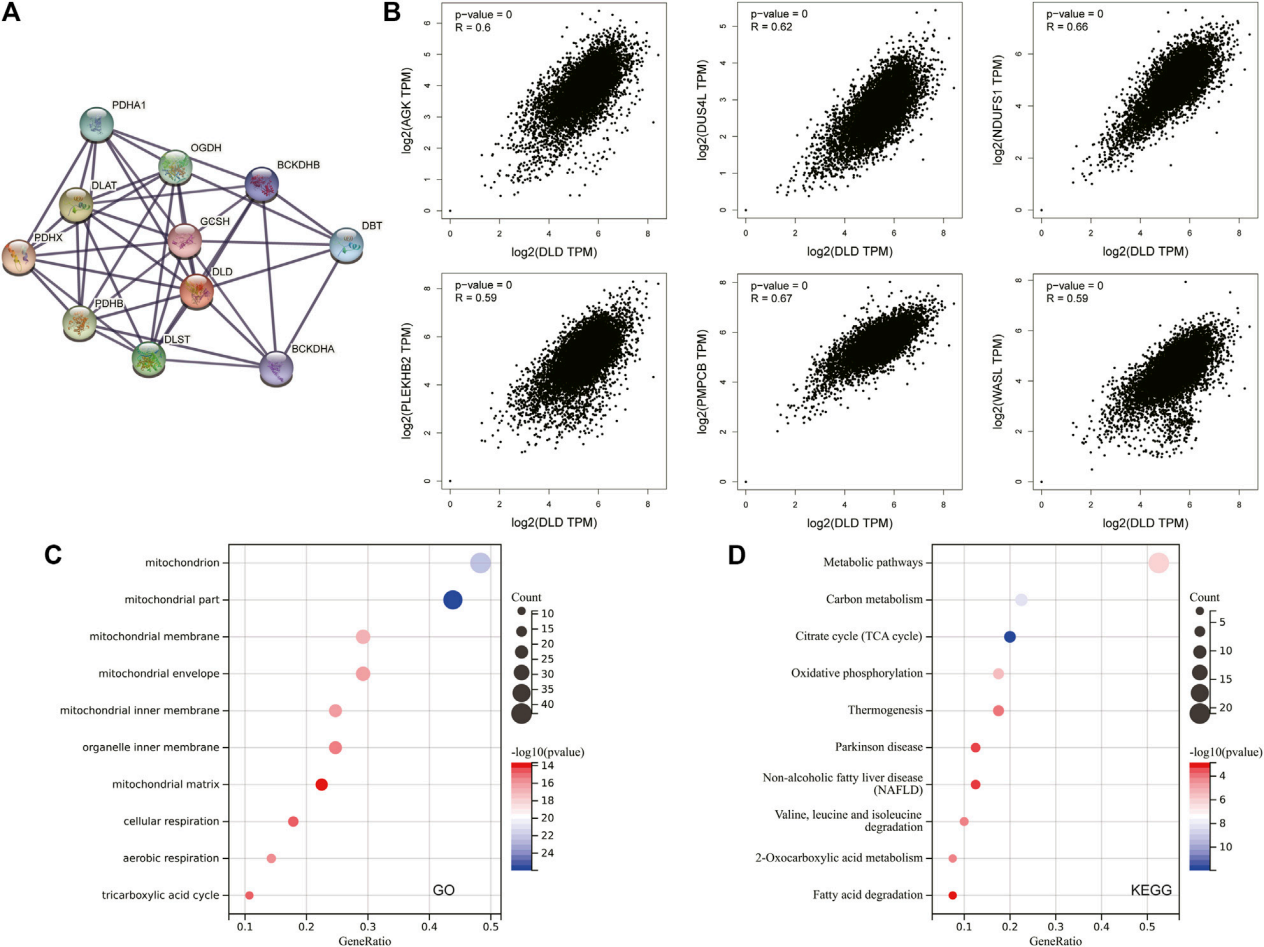
DLD-related gene enrichment analysis. **(A)** The DLD-interacting molecular network generated with the STRING tool. **(B)** Correlation scatterplot of the top 6 genes associated with DLD obtained with the GEPIA2.0 tool. **(C)** GO enrichment analysis of DLD-related genes. **(D)** KEGG enrichment analysis of DLD-related genes.

To explore the potential molecular mechanisms involving DLD in tumorigenesis and progression, we further obtained the DLD-interacting molecular network by using the STRING tool (Figure 12A). Then, the top 100 genes associated with DLD expression in pancancer data were acquired, and the top 6 were selected to draw the scatter plot in Figure 12B. Finally, GO and KEGG enrichment analyses were performed on these top 100 DLD-related genes, and the top 10 results are shown in bubble plots. As shown in Figure 12C, GO enrichment analysis indicated that DLD-related genes were mainly associated with mitochondria-related cellular components, aerobic respiration and the tricarboxylic acid cycle. The pathways obtained by KEGG enrichment analysis are also displayed in Figure 12D.

### Correlations of DLD expression with immune checkpoint genes or immunoregulatory genes

**FIGURE 13:**
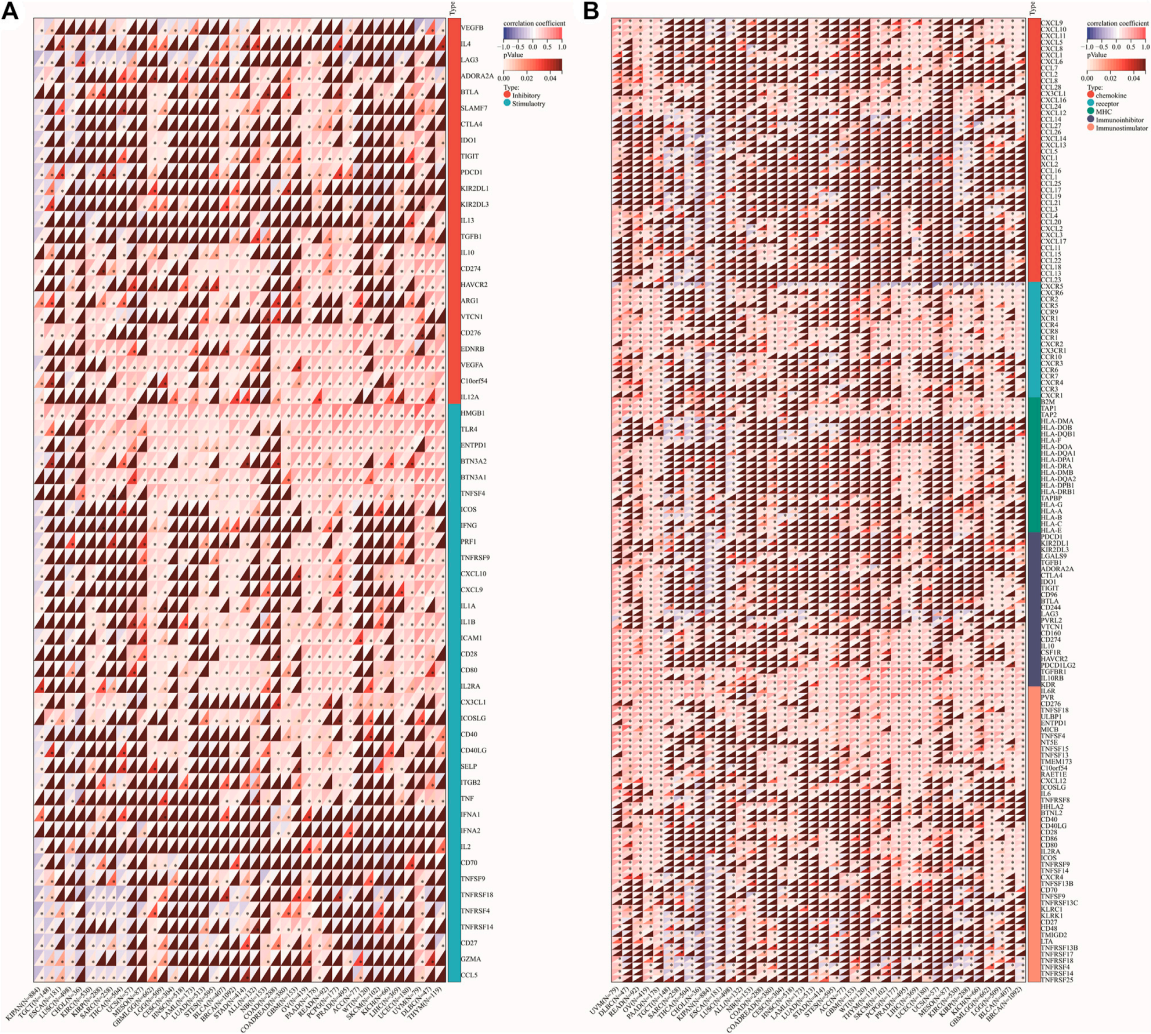
Correlation of DLD expression with immune checkpoint genes or immunoregulatory genes. **(A)** Immune checkpoint genes. **(B)** Immunoregulatory genes. *p < 0.05.

We explored the relationships between DLD expression and 60 two-class immune checkpoint pathway marker genes including inhibitory (24) and stimulatory (36) genes (Thorsson et al., 2018), as well as 150 five-class immune pathway marker genes including chemokine (41), receptor (18), MHC (21), immunoinhibitory (24), and immunostimulatory (46) genes (Hu et al., 2021). Our results showed that DLD expression was positively correlated with immune checkpoint- and immunomodulatory activity-related genes in most tumors (Figures 13A-B).

### Relationship between DLD expression and drug sensitivity

**FIGURE 14:**
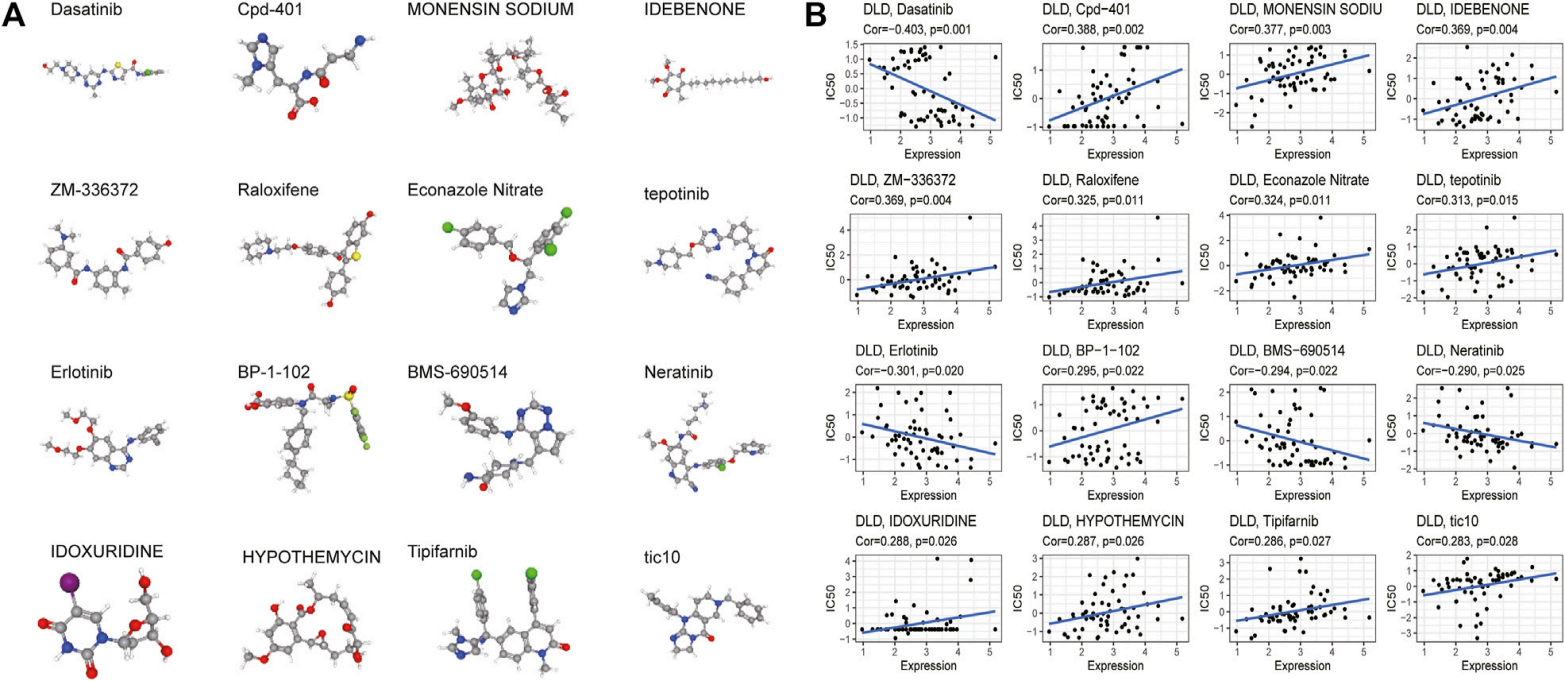
Drug sensitivity analysis. **(A)** 3D structure of the screened drugs. **(B)** Relationships between the DLD gene and sensitivity to antitumor drugs.

Finally, CellMiner data were used to explore the relationship between the DLD gene and sensitivity to common antitumor drugs. The correlation between DLD expression and the IC50 value of a drug was calculated by Pearson correlation analysis, and we obtained 33 significantly correlated drugs (*p-value* < 0.05). The results are summarized in Supplementary Table S8, and the top 16 drugs were selected for plotting in scatter plots (Figure 14B). Our results indicated that DLD expression was negatively correlated with dasatinib, erlotinib, BMS-690514, and neratinib sensitivity and positively correlated with Cpd-401, MONENSIN SODIUM, IDEBENONE, ZM-336372, raloxifene, econazole nitrate, tepotinib, BP-1-102, IDOXURIDINE, HYPOTHEMYCIN, tipifarnib, and tic10 sensitivity. Furthermore, PubChem (https://pubchem.ncbi.nlm.nih.gov/) was used to obtain the 3D structures of these 16 drugs (Figure 14A).

### Relationship between DLD expression and grade in gliomas

**FIGURE 15:**
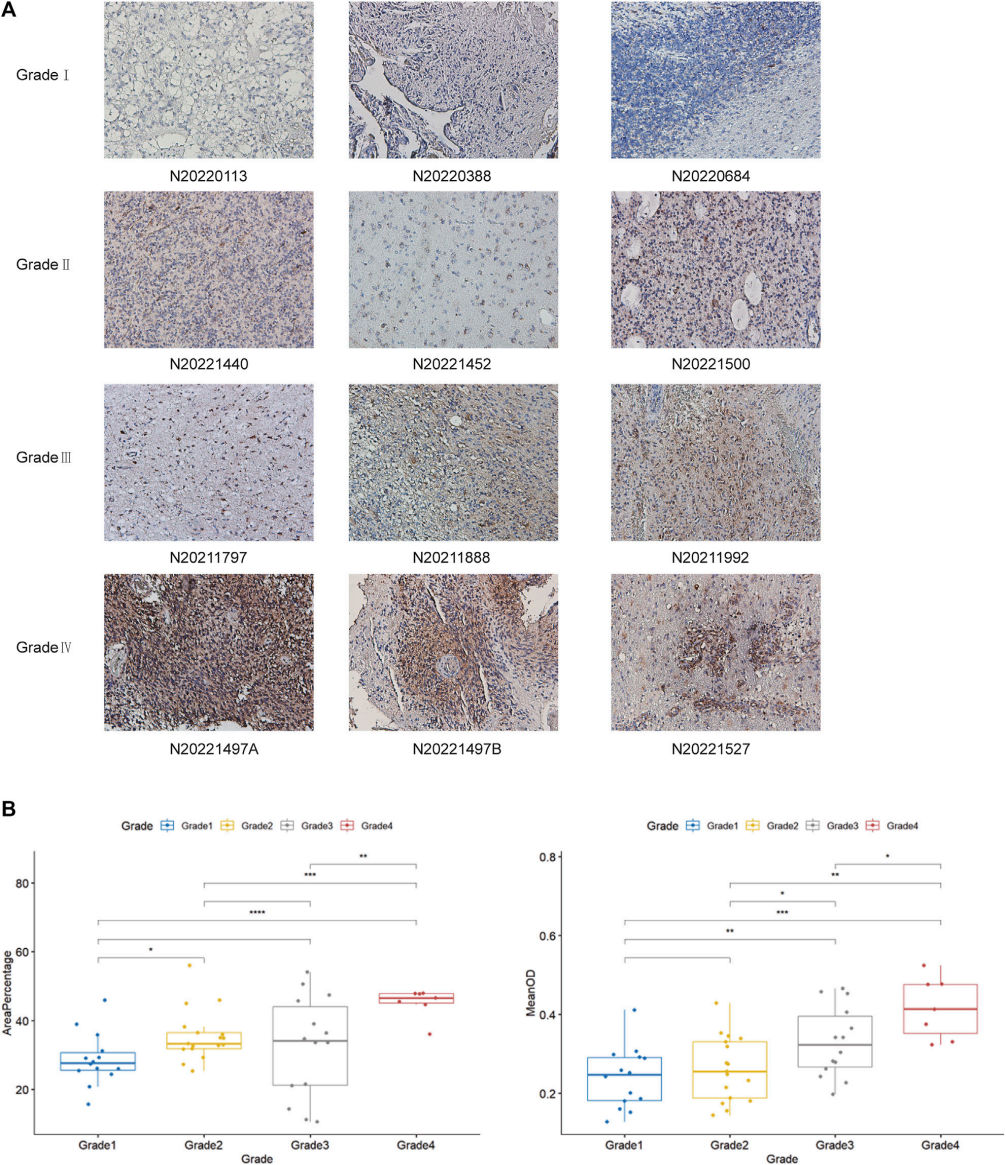
**(A)** Examination of DLD expression in each grade of glioma tissue by IHC analysis. **(B)** Analysis of DLD expression in each grade of glioma tissue by Student’s *t*-test. They include the percentage of positive area and The mean optical density values. ***p < 0.001; **p < 0.01; *p < 0.05.

By previous differential expression analysis of DLD, we found that the expression of DLD was higher in glioma tissues than in normal tissues. Then, the analysis of IHC results showed that DLD expression was significantly correlated with the grade of glioma (Figure 15A). The mean optical density values and the percentage of positive area responded to the expression of DLD in glioma tissues (Figure 15B). For the percentage of positive area, the results were statistically significant (*p*-value < 0.05), except for the comparison between grade I and grade III, grade II and grade III (*p*-value > 0.05). Similarly, for the mean optical density values, the results were statistically significant except for the comparison between grade I and grade II.

## Discussion

DLD, a positive regulator of cuproptosis, enhances copper-dependent cell death, which may provide new ideas for the therapeutic action of certain cancer-targeted drugs (Xiao et al., 2022). Previous studies have revealed that DLD is associated with several neurodegenerative diseases (Yan et al., 2008), and DLD deficiency may lead to several fatal metabolic diseases (Ambrus et al., 2016). Although the involvement of DLD in the constitutive pyruvate dehydrogenase complex (PDC) and α-ketoglutarate dehydrogenase complex (KGDC) has been identified as an important metabolic target in cancer (Kim et al., 2006; Semenza, 2010; Atlante et al., 2018), the role of DLD in the metabolism of tumor cells is less explored. Evidence from other studies has revealed that DLD downregulation may affect mitochondrial metabolism, which reduces the levels of downstream metabolites in the TCA cycle, induces melanoma cell death, and inhibits tumor progression in humans by promoting ROS production and altering energy metabolism (Yumnam et al., 2021). Tsvetkov et al. found that cuproptosis is a novel type of regulated cell death induced by copper ion carriers. Unlike apoptosis, ferroptosis, and necroptosis, cuproptosis relies on the regulation of mitochondrial respiration. Cuproptosis-related genes have been identified as prognostic biomarkers for a variety of cancers, such as UCEC, SKCM, and glioma (Chen, 2022; Lv et al., 2022; Wang et al., 2022). The results from a survival analysis showed that elevated DLD expression was associated with poor OS in patients with BRCA, KICH, LUAD or OV. The opposite results were obtained for COAD, KIRC and KIRP.

Immune cells play an important role in the composition of the tumor stroma. Complex interactions between cancer cells and immune cells lead to tumor growth and metastasis (Hinshaw and Shevde, 2019). The main site of interaction between tumor cells and immune cells is the tumor microenvironment (Nagarsheth et al., 2017). The response of tumor cells to immune checkpoint blockade (ICB) is associated with the tumor microenvironment, and ICB mainly exerts an effective antitumor effect through immune cell infiltration (Petitprez et al., 2020). Neutrophils are diverse and plastic cells that play key roles in resistance to infection and regulation of immunity. These cells can promote tumorigenesis by promoting angiogenesis, extracellular matrix remodeling, and immune suppression. Conversely, neutrophils can mediate antitumor responses by regulating tumor-resistant cell networks and killing tumor cells (Jaillon et al., 2020). Therefore, we evaluated the association of DLD with immune cell infiltration, immune checkpoint genes, and immunoregulatory genes. Interestingly, our findings showed that DLD expression was positively correlated with neutrophils, immune checkpoint genes, and immunomodulatory activity-related genes in most cancers. Thus, DLD may serve as a prognostic marker and potential target to improve immunotherapy in these tumors.

The heterogeneity of cancer cells poses a major challenge in the diagnosis and treatment of tumors, such as variations in proliferative activity, invasion and metastasis, and some cancer cells have stem cell-like properties (Patel et al., 2014; Giustacchini et al., 2017; Venteicher et al., 2017). Single-cell sequencing techniques can explore the multiple functional states of cancer cells (including status of stemness, invasion, metastasis, proliferation, EMT, angiogenesis, apoptosis, cell cycling, differentiation, DNA damage, DNA repair, hypoxia, inflammation and quiescence) (Yuan et al., 2019). Tirosh et al. found that cancer cells with high stemness may promote cancer progression (Tirosh et al., 2016). A study on glioblastoma also found heterogeneity in cancer-related functional states, such as the status of cell stemness, hypoxia, and proliferation (Yuan et al., 2019). Our study found that the expression of DLD was mainly positively correlated with some functional states of cancer cells. However, the opposite was found in UM. This suggests that DLD may influence the tumor developmental process by affecting the functional state of cancer cells.

Currently, there are few studies on the association of DLD with antitumor drug resistance in chemotherapy. Our results showed that DLD expression was correlated with tumor sensitivity to various drugs, such as dasatinib, erlotinib, BMS-690514, and neratinib. This finding suggests that DLD could also be a potential target molecule and predictor of cancer chemotherapy outcome. However, as a pure bioinformatic analysis that relies on data obtained from open databases, this should be confirmed by further investigation.

## Conclusion

In this study, the expression, survival impact, and genetic alterations of DLD across cancers were comprehensively analyzed by using bioinformatic techniques. DLD expression at the single-cell level and its role in the biological behavior of tumors were also investigated. In conclusion, DLD could be regarded as a novel biomarker for predicting patient prognosis in several types of tumors. Additionally, our results will help in the discovery of the potential roles of DLD in tumorigenesis and progression.

## Data Availability

The original contributions presented in the study are included in the article/Supplementary Material, further inquiries can be directed to the corresponding authors.

## Glossary

ACC: Adrenocortical Carcinoma
AML: Acute Myeloid Leukemia
BLCA: Bladder Urothelial Carcinoma
BRCA: Breast Invasive Carcinoma
CESC: Cervical Squamous Cell Carcinoma and Endocervical Adenocarcinoma
CHOL: Cholangiocarcinoma
COAD: Colon Adenocarcinoma
DLBC: Lymphoid Neoplasm Diffuse Large B-cell Lymphoma
ESCA: Esophageal Carcinoma
GBM: Glioblastoma Multiforme
HNSC: Head and Neck Squamous Cell Carcinoma
KICH: Kidney Chromophobe
KIRC: Kidney Renal Clear Cell Carcinoma
KIRP: Kidney Renal Papillary Cell Carcinoma
LAML: Acute Myeloid Leukemia
LGG: Brain Lower Grade Glioma
LIHC: Liver Hepatocellular Carcinoma
LUAD: Lung Adenocarcinoma
LUSC: Lung Squamous Cell Carcinoma
MESO: Mesothelioma
OV: Ovarian Serous Cystadenocarcinoma
PAAD: Pancreatic Adenocarcinoma
PCPG: Pheochromocytoma and Paraganglioma
PRAD: Prostate Adenocarcinoma
RB: Retinoblastoma
READ: Rectum Adenocarcinoma
SARC: Sarcoma
SKCM: Skin Cutaneous Melanoma
STAD: Stomach Adenocarcinoma
TGCT: Testicular Germ Cell Tumor
THCA: Thyroid Carcinoma
THYM: Thymoma
UCEC: Uterine Corpus Endometrial Carcinoma
UCS: Uterine Carcinosarcoma
UVM(UM): Uveal Melanoma.
