# Supplementary figures and images for: Comprehensive analysis of the cuproptosis-related gene DLD across cancers: A potential prognostic and immunotherapeutic target
Source: Front Pharmacol. 2023 Apr 3;14:1111462. doi: 10.3389/fphar.2023.1111462 (PMC10127393; doi:10.3389/fphar.2023.1111462)

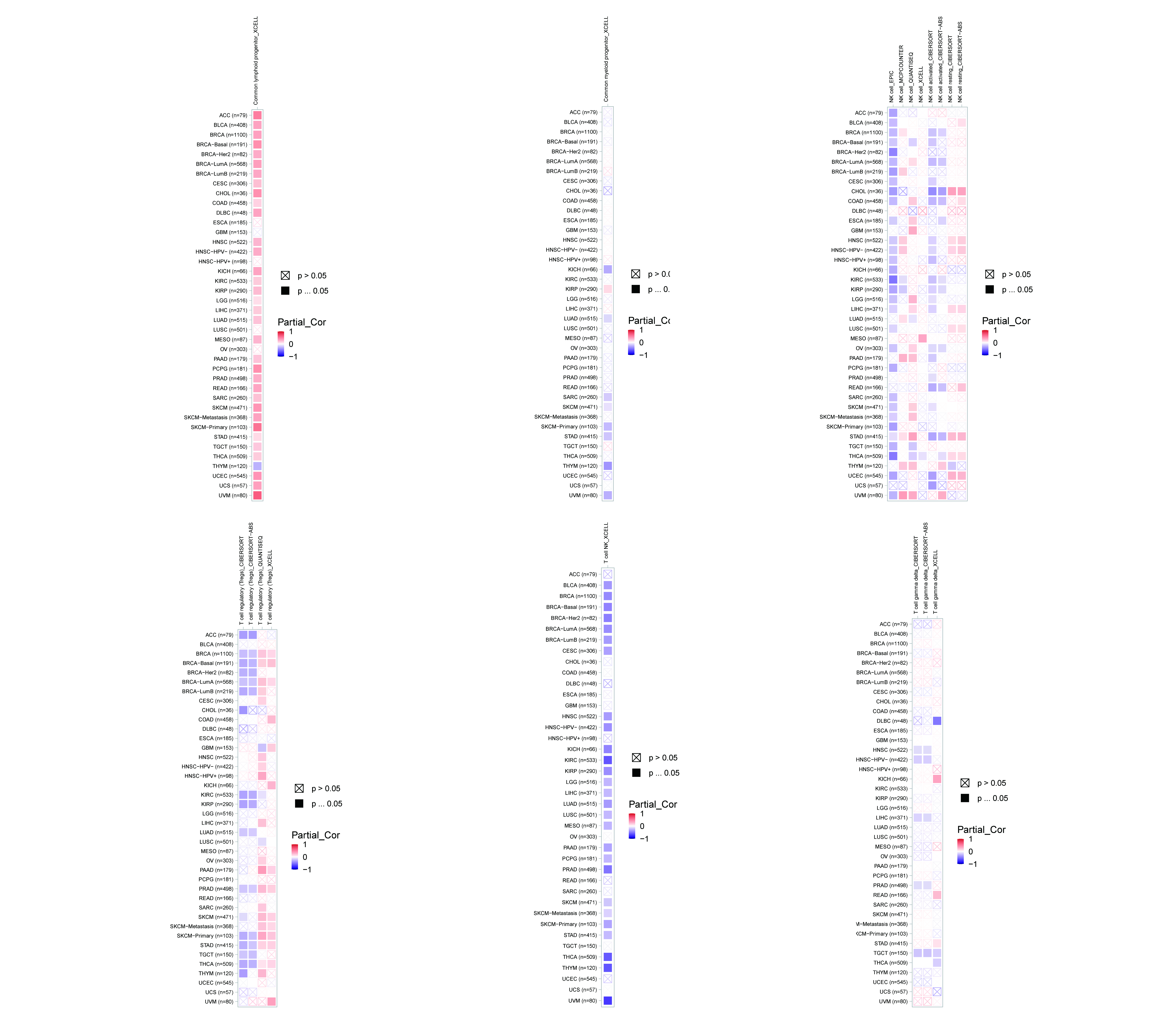

Supplement: Supplementary file 1 [file Image3.TIF]
